# Improving the diagnosis of endometrial cancer in postmenopausal women in primary care settings using an artificial intelligence-based ultrasound detecting model

**DOI:** 10.3389/fonc.2025.1646826

**Published:** 2025-09-09

**Authors:** Nan Wang, Ruoxi Zhang, Ling Dong, Ganjun Zhang, Shengnan Meng

**Affiliations:** ^1^ Department of Gynecology, Hospital of Traditional Chinese Medicine of Qiqihar, Qiqihar, China; ^2^ Department of Medicine, Intracardiac Echocardiography (ICE) Intelligent Healthcare, Soochow, China; ^3^ Department of Artificial Intelligence, Shanghai Bingzuo Jingyi Technology, Shanghai, China

**Keywords:** endometrial cancer, gynecological ultrasound, primary care settings, artificial intelligence, deep learning

## Abstract

**Objectives:**

We aimed to develop a deep learning (DL) model based on ultrasound examination to assist in ultrasound-based assessment of confirmed endometrial cancer (EC) in postmenopausal women, with the goal of improving diagnostic efficiency for EC in primary care settings.

**Methods:**

A novel DL system was developed to analyze comprehensive gynecological ultrasound images, specifically targeting the identification of EC based on ultrasound features, using the diagnosis made by ultrasound specialists as the reference standard. Ultrasound measurements were performed to assess endometrial thickness and tumor homogeneity in all patients using gray-scale sonography. Intertumoral blood flow characteristics were analyzed through the blood flow area (BFA), resistance index (RI), end-diastolic velocity (EDV), and peak systolic velocity (PSV). The system’s performance was assessed using both internal and external test sets, with its effectiveness evaluated based on agreement with the ultrasound specialist and the area under the receiver operating characteristic (ROC) curve for binary classification.

**Results:**

A total of 877 patients with EC diagnosed by endometrial biopsy at Hospital of Traditional Chinese Medicine of Qiqihar between January 1, 2020, and December 31, 2024, were enrolled in this study. 877 ultrasound images were divided into three groups: 614 for training, 175 for validation, and 88 for testing. The AUC for the training set was 0.844 (95% CI: 0.784–0.893). In the validation set, the AUC for predicting EC was 0.811 (95% CI: 0.748-0.864), while in the testing set, the AUC reached 0.858 (95% CI: 0.800-0.905).

**Conclusions:**

The DL model demonstrated high accuracy and robustness, significantly enhancing the ability to diagnostic assistance for EC through ultrasound in postmenopausal women. This provides substantial clinical value, especially by enabling less experienced physicians in primary care settings to effectively detect EC lesions, ensuring that patients receive timely diagnosis and treatment.

## Introduction

Endometrial cancer (EC) is the most common gynecological malignancy in developed countries, with increasing incidence rates worldwide (1). It typically affects postmenopausal women and is associated with risk factors such as obesity, diabetes, and hormone imbalances (1). Currently, gynecological ultrasound remains the primary detecting method for endometrial cancer due to its non-invasive nature, cost-effectiveness, and relatively high diagnostic efficacy (2). Gynecological ultrasound allows for the assessment of endometrial thickness and morphology, providing valuable information for early detection and diagnosis (3).

However, the implementation of effective ultrasound detecting in primary care settings faces several challenges. These include insufficient diagnostic expertise among primary care physicians, inadequate ultrasound operation skills, and limited access to high-quality imaging equipment (3, 4). Furthermore, the interpretation of ultrasound findings can be subjective and requires experience, particularly in distinguishing between benign and malignant conditions (5). These factors may lead to potential misdiagnoses or delayed referrals to specialists, highlighting the need for ongoing training and standardization of ultrasound protocols in primary care facilities to improve the early detection and management of endometrial cancer (6).

Artificial intelligence (AI) encompasses a wide range of computational methods designed to replicate logical, often human-like, reasoning. One of the most impactful subsets of AI is deep learning (DL), which leverages multiple layers of processing to tackle complex tasks, such as analyzing images and medical data [7]. When trained on large, detailed datasets for specific applications, deep learning (DL) models can outperform even highly skilled human experts in terms of accuracy (7). Currently, the application of AI in the field of modern medicine is already very extensive, primarily in areas such as image recognition, diagnostic assistance, and disease risk modeling (8). Based on the aforementioned factors, we aim to develop a DL model based on ultrasound examination to identify EC in postmenopausal women, with the goal of improving diagnostic efficiency for EC in primary care settings.

## Methods

### Ethical statement

The study was approved by the ethics committee of the Hospital of Traditional Chinese Medicine of Qiqihar with ethical approval number KY2023-108, and was conducted in accordance with the Declaration of Helsinki and Good Clinical Practice Guidelines. All patients provided written informed consent prior to inclusion in the study. The consent process was conducted in accordance with institutional and national guidelines, ensuring that participants were fully informed of the study purpose and data usage. Furthermore, all ultrasound image data were de-identified prior to analysis to protect patient privacy, and only anonymized datasets were used for model training and evaluation. No personally identifiable information was stored or processed during this study.

### Data sources and study population

A prospective study was conducted on 877 postmenopausal patients diagnosed EC by endometrial biopsy at Hospital of Traditional Chinese Medicine of Qiqihar between January 1, 2020, and December 31, 2024. Postmenopausal women with confirmed EC of any histologic subtype were eligible for enrollment. Patients were excluded if they had recurrent EC. Key clinical characteristics such as patient age, years since menopause, and major comorbidities (e.g., hypertension, diabetes) were collected at baseline. This study only included patients with histologically confirmed EC; no benign or normal control cases were included in the dataset.

**Figure 1:**
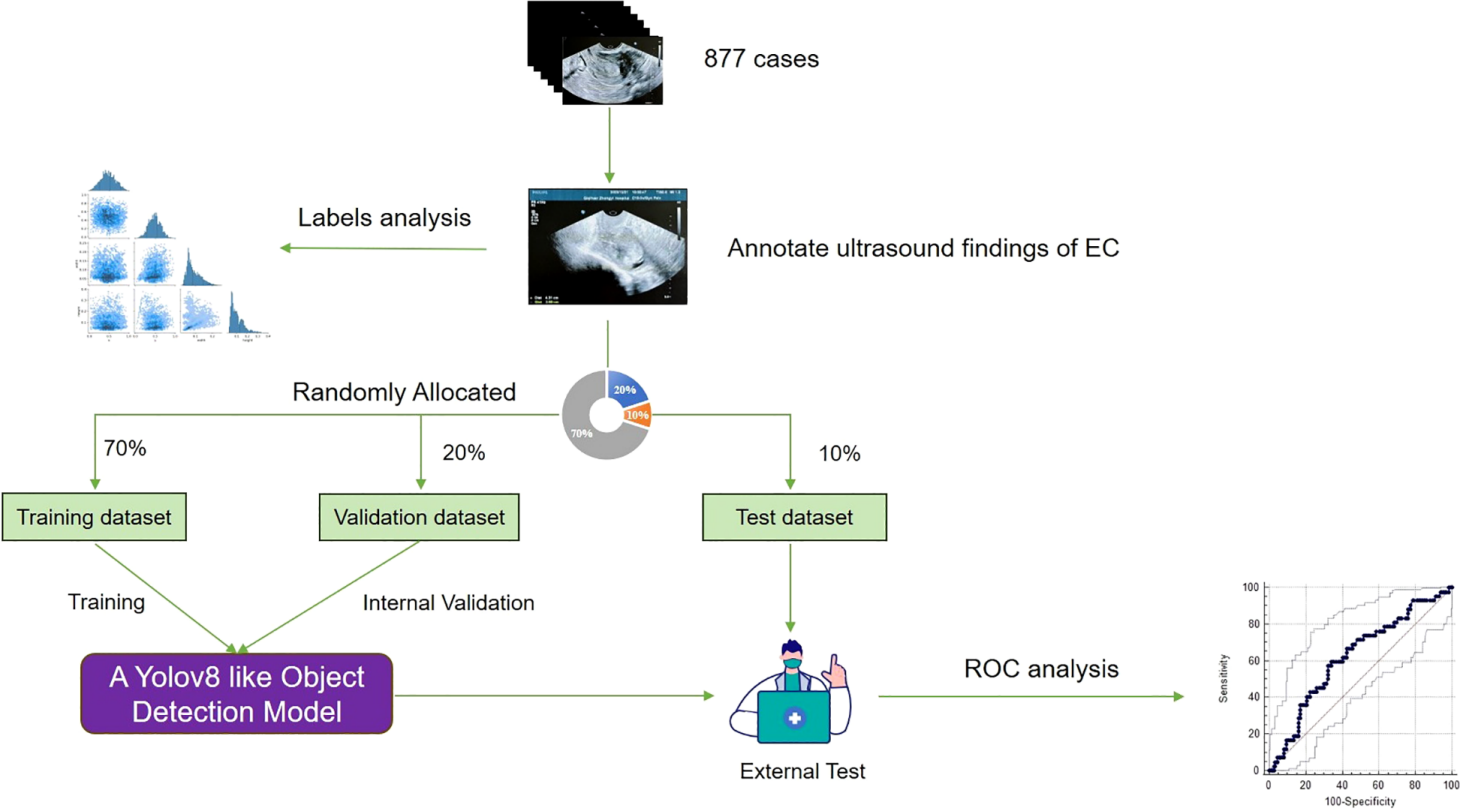
Study flowchart.

In order to train and validate the DL model, patients were randomly divided into the training set (n = 614), the validation set (n = 175), and the testing set (n = 88) in a 7:2:1 ratio (**Figure 1**). The training set was used to train the neural network, the validation set was used to optimize the network and select the parameters, and the testing set was used to evaluate the performance of the neural network.

### Endometrial biopsy

For safety purposes, endometrial biopsies were mandatory before participants could enter the study. These biopsies were performed by clinical staff at the Hospital of Traditional Chinese Medicine in Qiqihar and were analyzed by pathologists who were blinded to the random assignment.

### Conventional imaging evaluation and labeling

**Figure 2:**
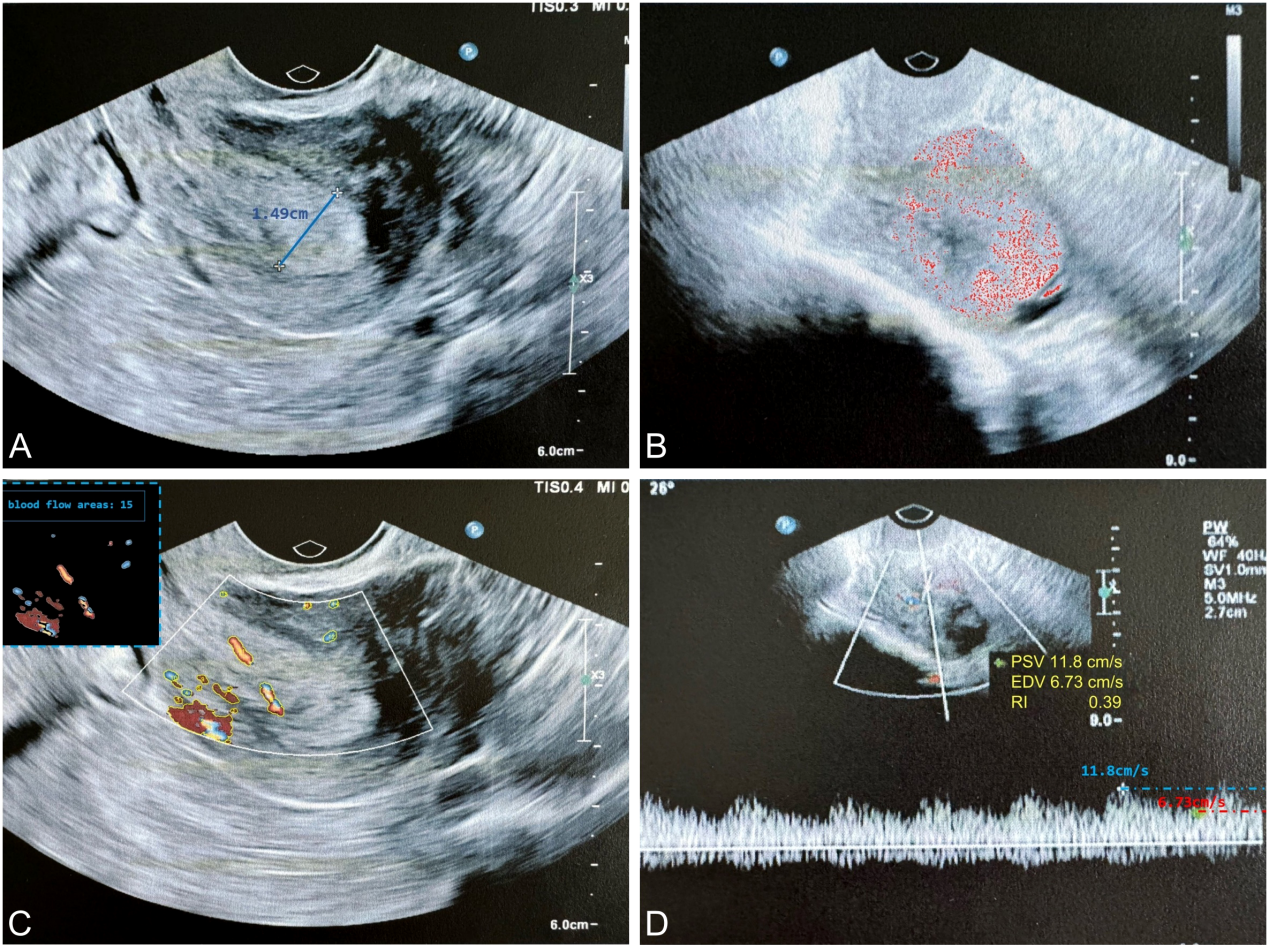
The typical ultrasound exploration image of EC highlights key diagnostic features. **(A)** The endometrial thickness was measured at 1.49 cm (thickness ≥ 0.5 cm suggests a higher probability of malignancy); **(B)** Ultrasound examination revealed heterogeneous echogenicity within the tumor, suggesting a higher probability of malignancy; **(C)** Color Doppler imaging demonstrated increased vascularity within the tumor, characterized by a punctate or arborescent distribution pattern, suggesting a higher probability of malignancy; **(D)** Spectral Doppler analysis revealed a RI < 0.4, indicative of neoangiogenesis. EC, endometrial cancer; RI, resistance index.

The ultrasound examination was performed at the Hospital of Traditional Chinese Medicine in Qiqihar, which is one of the top medical centers in the northwest region of Heilongjiang Province, China. Regarding image heterogeneity, all ultrasound images were obtained using standardized scanning protocols at a single institution. Two experienced ultrasound specialists performed a consensus reading of all available ultrasound imaging and clinical information for each patient to establish the reference standard, including thickening of the tumor tissue endometrium, abnormal ultrasound echoes, and abnormal blood flow signals (**Figure 2**).

Based on this reference standard, one specialist manually annotated the tumor regions in each ultrasound image slice by drawing bounding rectangles that fully encompassed the tumor. In addition, the radiologist also labeled each tumor as benign or malignant, based on the corresponding pathological diagnosis. To ensure annotation accuracy and minimize inter-observer variability, another specialist with expertise in ultrasound imaging independently reviewed the annotations and refined them as needed. The refined annotations were then adopted as the ground truth (9, 10).

To further mitigate device- or operator-related variability, all images were resized to a uniform input resolution with preserved aspect ratio and pixel intensities were normalized to a standardized scale. The annotation process ensured consistent labeling despite the inherent image variability across patients, allowing the DL model to learn diagnostic features representative of real-world clinical heterogeneity. By incorporating expert-validated annotations, the model was effectively trained to generalize across variations in image quality, anatomical differences, and scanning conditions.

In addition, during training, we applied multiple data augmentation techniques—such as brightness/contrast perturbation, affine transformations, and noise injection—to simulate real-world imaging variation and improve the model’s generalizability.

### Development of the DL algorithm

Object detection is a fundamental task in DL that involves both the localization and classification of objects present in images (11). In this study, we adopted YOLOv8 (12), a state-of-the-art one-stage object detection framework, consisting of backbone, neck, and YOLO head. The backbone, also known as the feature extractor, is crucial for extracting meaningful features from the input image. The neck serves as a bridge between the backbone and the head, performing feature fusion operations and integrating contextual information. The YOLO head is the final part of the network and is responsible for generating the outputs, such as bounding boxes and confidence scores for object detection. YOLOv8 offers several advantages that make it well-suited for real-time clinical applications, including its efficient anchor-free design, decoupled head for classification and regression, and improved feature aggregation via a streamlined backbone and neck structure. In clinical practice, radiologists determine the location of a tumor by identifying regions that exhibit differences in echogenicity, tissue density, and vascular patterns compared to the surrounding normal tissue. From a computer vision perspective, tumors often present with distinctive structural and morphological characteristics that differentiate them from healthy tissue. Moreover, the assessment of tumor malignancy is commonly based on features such as thickening of adjacent tissue, abnormal internal echo patterns, and irregular or increased blood flow signals observed on Doppler imaging. These imaging cues play a critical role in clinical diagnosis. Given the limited availability of annotated clinical ultrasound data, it becomes essential to effectively leverage these domain-specific features to enhance model accuracy and align the prediction process with radiological reasoning.

**Figure 3:**
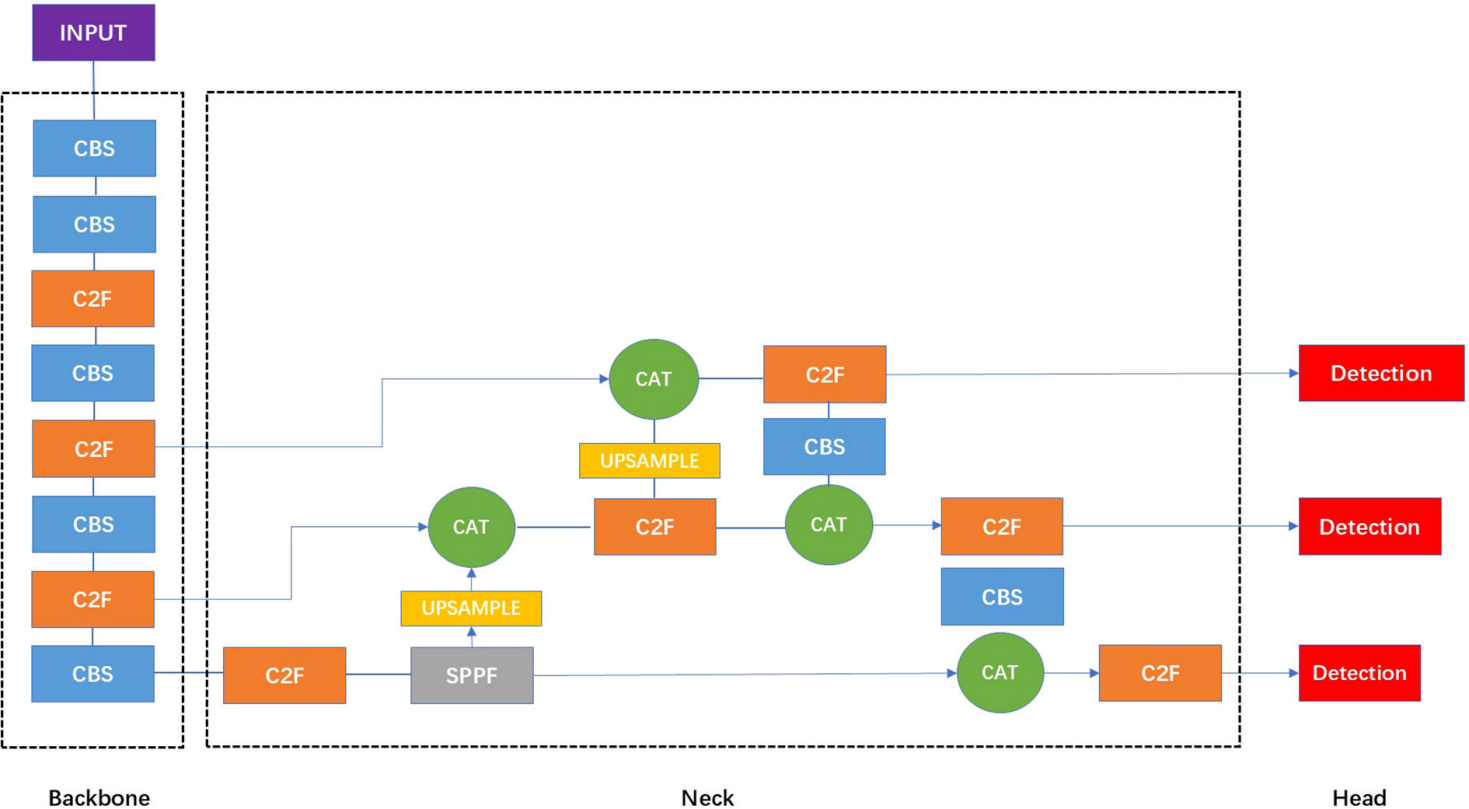
The structure of the standard YOLOv8 network. In the architecture visualization of YOLOv8, the CBS module is a fundamental building block that sequentially applies a convolutional layer, batch normalization, and the SiLU activation function, serving as a standard feature extraction unit. The C2F module integrates high-level features with contextual information to enhance detection accuracy. The SPPF layer refers to spatial pyramid pooling faster module which is designed to expand the receptive field without additional downsampling. The Upsample layers are used to increase the resolution of the feature maps. The CAT module refers to a concatenation operation that merges feature maps from different layers or scales, facilitating multi-level feature integration for enhanced representation learning.

Firstly, to improve the model’s adaptability to tumor shape variability and enhance its capacity to capture complex morphological features, we incorporated deformable convolutional layers (13) into the YOLOv8 framework. Deformable convolutions allow dynamic adjustment of sampling positions within the convolutional kernel, enabling more flexible and precise feature extraction from irregular or non-rigid structures, which are common characteristics of tumors in ultrasound images. Unlike standard convolutions, deformable convolutions introduce learnable offsets to each sampling location within the convolutional kernel, enabling the kernel to adapt dynamically to the geometric variations of the target object. This flexibility allows the network to better capture irregular shapes and structural deformations, which are common in medical and infrared imaging tasks. For instance, using a 3×3 convolutional kernel with a dilation rate of 1, the set of standard sampling positions is defined as


$$R\;=\left\{\left(-1,-1\right),\left(-1,0\right),\ldots ,\left(0,1\right),\left(1,1\right)\right\}$$

where each element represents the relative offset of a sampling point with respect to the kernel center. In deformable convolutions, these offsets are learnable and data-dependent, enabling the model to effectively learn multiple optimal sampling configurations across varying object scales and shapes. The overall architecture of the proposed model follows the original YOLOv8 framework (**Figure 3**). We replaced standard convolutions with deformable convolutions at key locations in the backbone. Specifically, after the second and third C2F modules, where high-level semantic and spatially variant features are extracted. Other components, including the initial CBS modules, the SPPF layer, the feature fusion neck, and the detection head, were retained to preserve the model’s efficiency and scalability.

Secondly, to address the issue of tumor tissue being easily confused with structures like uterine muscular and benign tissue, we optimized the detection pipeline to improve differentiation accuracy. We applied Mosaic data augmentation, which involves randomly scaling, cropping, and arranging four images to create composite images. This approach increases the diversity of tumor tissue samples in the detection dataset, and the random scaling introduces a variety of small features, thereby improving the robustness of the detection process. Additionally, we designed a dynamic prototype loss function to better distinguish tumor tissue from other similar structures. The formula is as follows,


$$L_{DPL}=-\log\frac{e^{{\tilde{W}}_{y_{i}}^{T}x_{i}}}{e^{{\tilde{W}}_{y_{i}}^{T}x_{i}}+\sum_{j=1,\neq y_{i}}^{N}e^{{\tilde{W}}_{j_{i}}^{T}x_{i}}}$$

where $W_{j}\in {\mathbb{R}}^{d}$ denotes the j-th  column of class-wise prototype $W\in {\mathbb{R}}^{d\times N}$ , d is the feature dimension, N is the class number, and $x_{i}\in {\mathbb{R}}^{d}$ denotes the feature of the i-th  sample, belonging to the $y_{i}\text{-th }$ class. The variational prototype $\tilde{W}$ is sampled from the class-wise distribution W , and T denotes the transpose of a matrix.

YOLOv8 employs a composite loss function that includes classification loss, bounding box regression loss, and distributional localization loss. Specifically, the classification loss is computed using Varifocal Loss (VFL) (14), the regression loss is based on Complete Intersection over Union (CIoU) (15), and the distributional component is modeled using Distribution Focal Loss (DFL). These three loss components are combined using predefined weight coefficients. $L_{DPL}$ automatically aggregates non-tumor target structures, reducing intra-class distance for the tumor category while increasing inter-class distance between tumor and other similar structures. In our implementation, the original DFL was replaced by $L_{DPL}$ to improve localization accuracy and enhance discrimination between tumor and non-tumor regions.

The overall loss function combines three components through weighted summation: the CIoU loss for localization, the dynamic prototype loss, and the VFL for confidence loss of object bounding boxes.


$$L_{total}=\;\lambda_{loc}L_{CIoU}+\lambda_{conf}L_{VFL}+\lambda_{cls}L_{DPL}$$

where $\lambda_{loc}$ , $\lambda_{conf}$ , and $\lambda_{cls}$ are weight coefficients used to balance the contributions of different loss terms. These losses work collaboratively to identify tumor structures. This combination of data augmentation and a specialized loss function led to a significant improvement in the precision of tumor identification, ensuring more accurate detection and reducing false positives in ultrasound images.

### Ablation analysis

**Figure 4:**
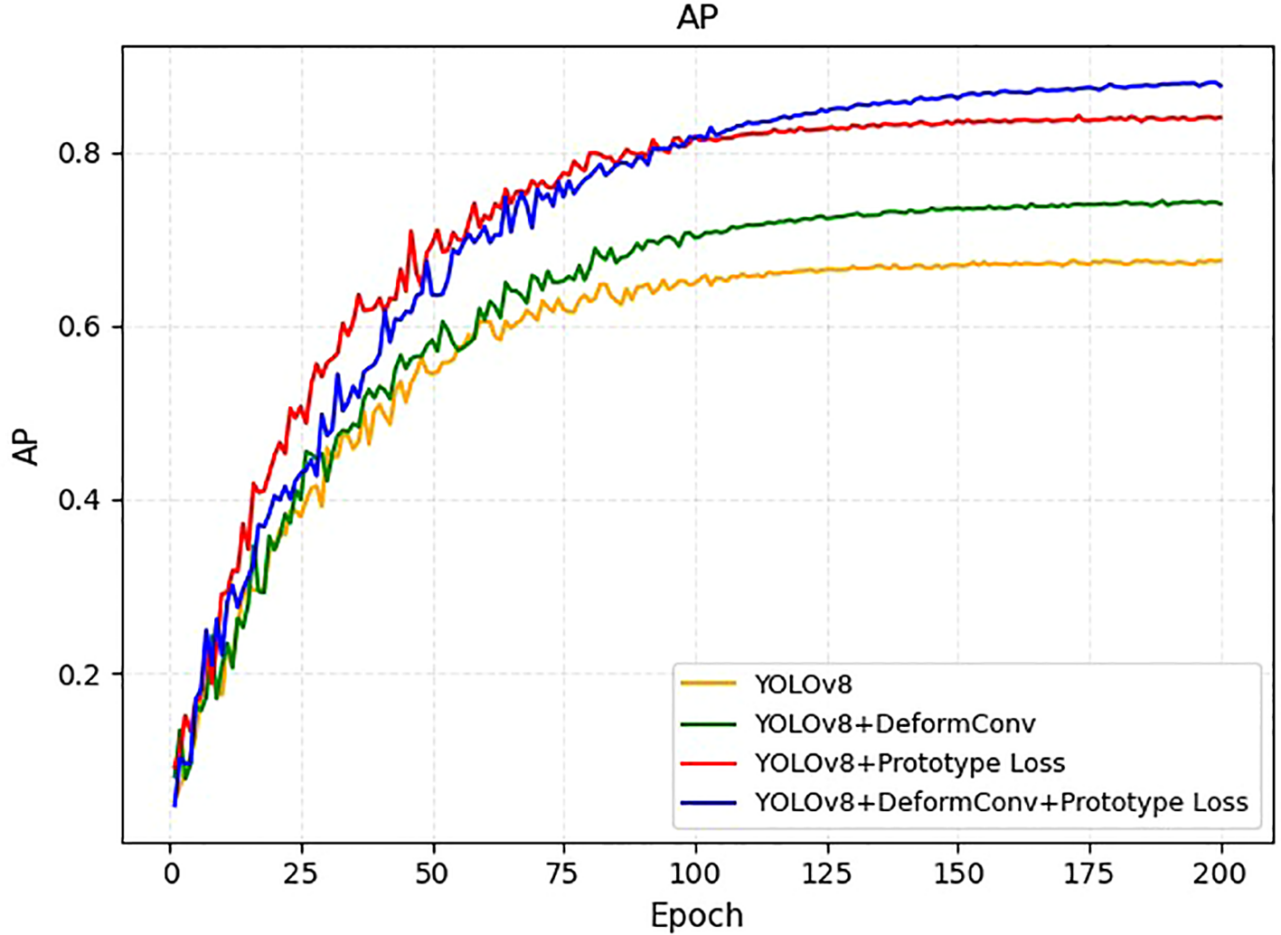
AP learning curves on the training set for four model variants: YOLOv8 (orange), YOLOv8+DeformConv (green), YOLOv8+Prototype Loss (red), and YOLOv8+DeformConv+Prototype Loss (blue).

To evaluate the individual contributions of the deformable convolutions and prototype loss components, we conducted an ablation experiment during the training process and present the results as Average Precision (AP) curves (**Figure 4**). Starting from the baseline YOLOv8 model, we observed that incorporating deformable convolution alone improved performance throughout the training process and resulted in a higher final AP compared to the baseline. Similarly, introducing the prototype loss function to the YOLOv8 framework accelerated convergence in the early epochs and provided a noticeable gain in the final detection accuracy. Notably, combining both deformable convolution and prototype loss yielded the highest AP among all configurations, demonstrating a synergistic effect. These results validate the effectiveness of the proposed architectural improvements and highlight their respective and combined contributions to the overall model performance. As the dataset exclusively consisted of confirmed EC cases, the model cannot currently be used to differentiate EC from benign endometrial conditions in a primary screening population.

### Interpretability discussion

**Figure 5:**
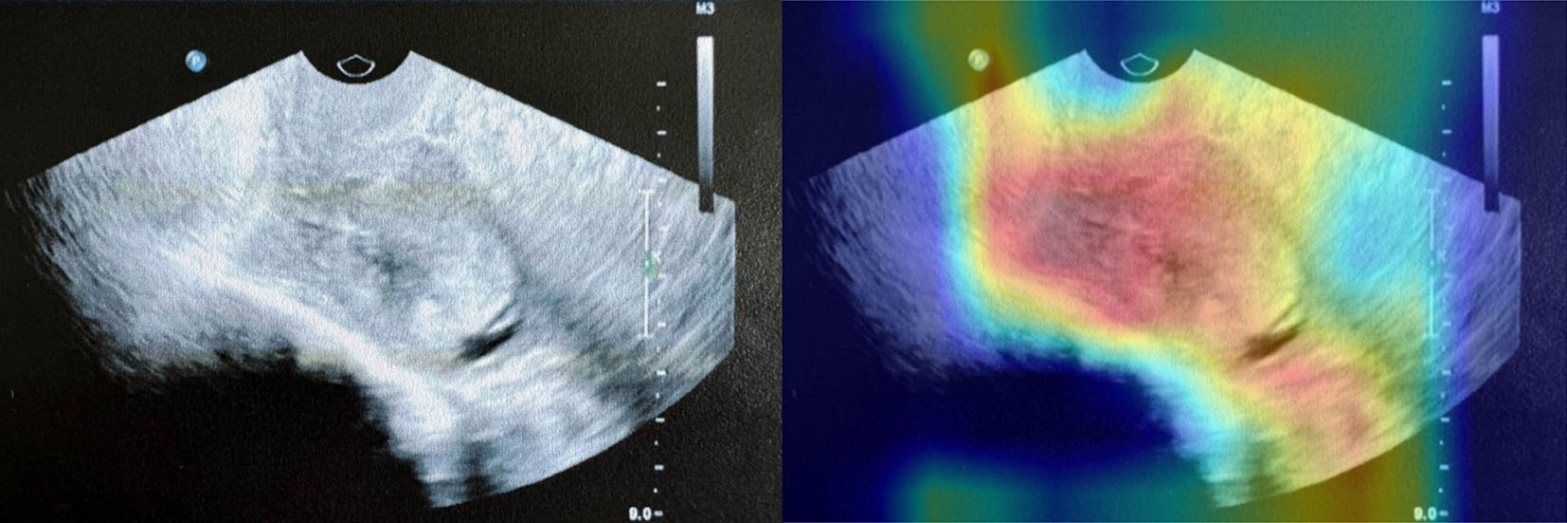
An example of Grad-CAM interpretability analysis in testing set. The left panel shows the original ultrasound image, while the right panel overlays the Grad-CAM heatmap, highlighting the regions to which the model paid the most attention during prediction.

To improve understanding of our detection results, we adopted Gradient-weighted Class Activation Mapping (Grad-CAM) (16) to visualize the spatial attention of the predictions. Grad-CAM generates heatmaps based on the gradients of class scores with respect to feature maps in the final convolutional layers, highlighting regions in the input image that contribute most strongly to the model’s predictions. As illustrated in our Grad-CAM heatmap (**Figure 5**), the original ultrasound image is compared with its corresponding Grad-CAM heatmap overlay. The Grad-CAM heatmap allows us to qualitatively assess whether the model focuses on clinically meaningful features, such as the endometrial lining or abnormal echogenic regions, without any explicit measurement information was provided as input.

### Statistical analysis

Quantitative variables were expressed as mean value ± standard deviation, and qualitative variables were expressed as total number and percentage. The independent two-sample t-test or one-way analysis of variance (ANOVA) with posthoc Student Newman-Keuls test was used to assess the differences between multiple sets of data. Categorical variables were also compared using the chi-square or Fisher’s exact test. Model performance was assessed in the test cohorts on their ultrasound imaging features, such as echo characteristics, tissue structure, resolution, and blood flow velocity. A weighted Kappa coefficient was calculated with the 4×4 contingency table comparing the model results with the ultrasound specialist’s clinical EC diagnosis with quadratic weighting to more significantly penalize model classifications that were highly discordant from the ultrasound specialist’s assessments. Specifically, the model prediction was considered correct when the predicted tumor type (benign or malignant) matched the ground truth, and the predicted tumor location had an intersection over union (IoU) greater than 0.5 with the expert-annotated bounding box. Otherwise, the prediction was regarded as incorrect. The receiver operating characteristic curve (ROC) was obtained by using Python 3.5, Matplotlib 3.0.2 and ROC module. The area under the ROC curve (AUC) values of test sets to analyze whether the models were robust, and we used the mean accuracy, sensitivity, and specificity of the ultrasound specialists to compare the performance of the deep learning algorithm. A 2-sided p-value < 0.05 was considered statistically significant.

## Results

### Basic characteristics

The screening process involved 1030 patients, among whom 877 were finally enrolled in the study between January 1, 2020, and December 31, 2024. The baseline demographic and clinical characteristics of patients with EC are summarized in **Table 1**, while **Table 2** delineates the distribution of EC subtypes observed in the study population. There were no significant differences in baseline characteristics among the training, validation, and testing sets.

**Table 1:** Baseline characteristics of postmenopausal women with endometrial cancer.

Characteristics	Training set (n=614)	Validating set (n=175)	Testing set (n=88)	*P*-value
Age (years)	69.8 ± 4.5	70.5 ± 4.8	70.6 ± 4.9	0.387
Years since menopause (years)	16.9 ± 3.2	17.3 ± 3.4	17.4 ± 3.3	0.079
BMI (kg/m^2^)	28.1 ± 6.0	28.7 ± 6.3	28.7 ± 6.3	0.192
Waist circumference (cm)	89.2 ± 8.1	90.1 ± 8.4	90.4 ± 8.4	0.308
History of diabetes, n (%)	59 (9.6)	19 (10.9)	9 (10.2)	0.884
History of hypertension, n (%)	237 (38.6)	71 (40.6)	36 (40.9)	0.844
Smoking status, n (%)				
Never	398 (64.8)	117 (66.9)	58 (65.9)	0.877
Former	216 (35.2)	58 (33.1)	30 (34.1)	0.877
Current	–	–	–	–
Ever pregnant, n (%)				
Yes	551 (89.7)	159 (90.9)	80 (90.9)	0.876
No	63 (10.3)	16 (9.1)	8 (9.1)	0.876
Ever used postmenopausal estrogen pills, n (%)				
Yes	149 (24.3)	45 (25.7)	23 (26.1)	0.880
No	465 (75.7)	130 (74.3)	65 (73.9)	0.880
Ever used postmenopausal progestin pills, n (%)				
Yes	54 (8.8)	17 (9.7)	8 (9.1)	0.932
No	560 (91.2)	158 (90.3)	80 (90.9)	0.932

Mean values (standard deviation) and % (n) were reported for continuous and categorical variables, respectively. BMI, body mass index.

**Table 2:** Type of endometrial cancer.

Characteristics	Training set (n=614)	Validating set (n=175)	Testing set (n=88)	*P*-value
Tumor stage, n (%)				
Local	499 (81.3)	145 (82.9)	73 (83.0)	0.850
Regional/distant	115 (18.7)	30 (17.1)	15 (17.0)	0.850
Tumor grade, n (%)				
Well differentiated	165 (26.9)	49 (28.0)	25 (28.4)	0.926
Moderately differentiated	220 (35.8)	66 (37.7)	33 (37.5)	0.877
Poorly differentiated/anaplastic	229 (37.3)	60 (34.3)	30 (34.1)	0.686
Tumor size, n (%)				
<2 cm	109 (17.8)	33 (18.9)	17 (19.3)	0.903
2-<3 cm	91 (14.8)	28 (16.0)	14 (15.9)	0.910
3-<4 cm	202 (32.9)	60 (34.3)	31 (35.2)	0.877
≥4 cm	212 (34.5)	54 (30.8)	26 (29.6)	0.485
Histology, n (%)				
Type I	500 (81.4)	145 (82.9)	73 (83.0)	0.877
Type II	28 (4.6)	8 (5.5)	4 (4.5)	1.000
Other	86 (14.0)	22 (11.6)	11 (12.5)	0.846

Mean values (standard deviation) and % (n) were reported for continuous and categorical variables, respectively.

### Training set

**Figure 6:**
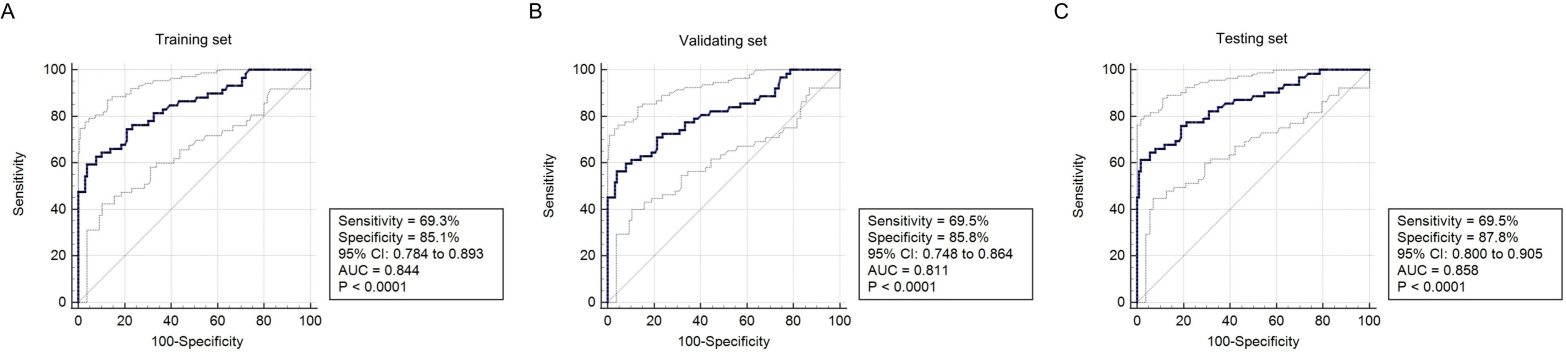
The predictive performance of DL model. DL, deep learning. **(A)** Training set; **(B)** Validating set; **(C)** Testing set.

A total of 614 ultrasound cases were used for training the DL model. The DL model was trained using 7,544 images derived from this set. The diagnostic accuracy, sensitivity, specificity, positive predictive value (PPV), and negative predictive value (NPV) of DL MODEL were 86.5%, 73.9%, 90.8%, 83.1%, and 88.3%, respectively, for EC (**Table 3**). The performance metrics for EC yielded an AUC of 0.844 (95% CI: 0.784–0.893, *P <*0.001) (**Figure 6A**). These results indicate excellent model performance in distinguishing EC, with high sensitivity and specificity achieved during training.

**Table 3:** Diagnostic performance of the AI model for EC in the training, validating, and testing set.

Characteristics	Training set (95% CI)	Validating set (95% CI)	Testing set (95% CI)	*P*-value
AUC	0.844 (0.784 - 0.893)	0.811 (0.748 - 0.864)	0.858 (0.800 - 0.905)	0.619
Accuracy	0.865 (0.767 - 0.963)	0.847 (0.749 - 0.945)	0.858 (0.760 - 0.956)	0.873
Sensitivity	0.739 (0.641 - 0.837)	0.762 (0.664 - 0.860)	0.773 (0.675 - 0.871)	0.748
Specificity	0.908 (0.810 - 1.006)	0.895 (0.797 - 0.993)	0.901 (0.803 - 0.999)	0.942
PPV	0.831 (0.733 - 0.929)	0.823 (0.725 - 0.921)	0.836 (0.738 - 0.934)	0.857
NPV	0.883 (0.785 - 0.981)	0.879 (0.781 - 0.977)	0.867 (0.769 - 0.965)	0.912

AI, artificial intelligence; EC, endometrial cancer; AUC, area under the curve; CI, confidence interval; PPV, positive predicted value; NPV, negative predicted value.

### Validation set

The validation set included 175 ultrasound cases. This set was utilized to internally validate the DL model, with 1,437 images assessed for performance. The diagnostic accuracy, sensitivity, specificity, positive PPV, and NPV of DL MODEL were 84.7%, 76.2%, 89.5%, 82.3%, and 87.9%, respectively, for EC (**Table 3**). The performance metrics for EC in the training set yielded an AUC of 0.811 (95% CI: 0.748–0.864, *P <*0.001) (**Figure 6B**). These metrics reflect consistent model performance across different datasets, confirming the robustness of the model in accurately identifying EC during validation.

### Testing set

The internal testing set contained 88 ultrasound cases. The model was tested with 722 images from this set. The diagnostic accuracy, sensitivity, specificity, positive PPV, and NPV of DL MODEL were 85.8%, 77.3%, 90.1%, 83.6%, and 86.7%, respectively, for EC (**Table 3**). The performance metrics for EC in the training set yielded an AUC of 0.858 (95% CI: 0.800–0.905, *P <*0.001) (**Figure 6C**). There were no significant differences in diagnostic performance metrics—including AUC, accuracy, sensitivity, specificity, PPV, and NPV—among the training, validation, and testing sets. The corresponding *P*-values were 0.619, 0.873, 0.748, 0.942, 0.857, and 0.912, respectively.

**Figure 7:**
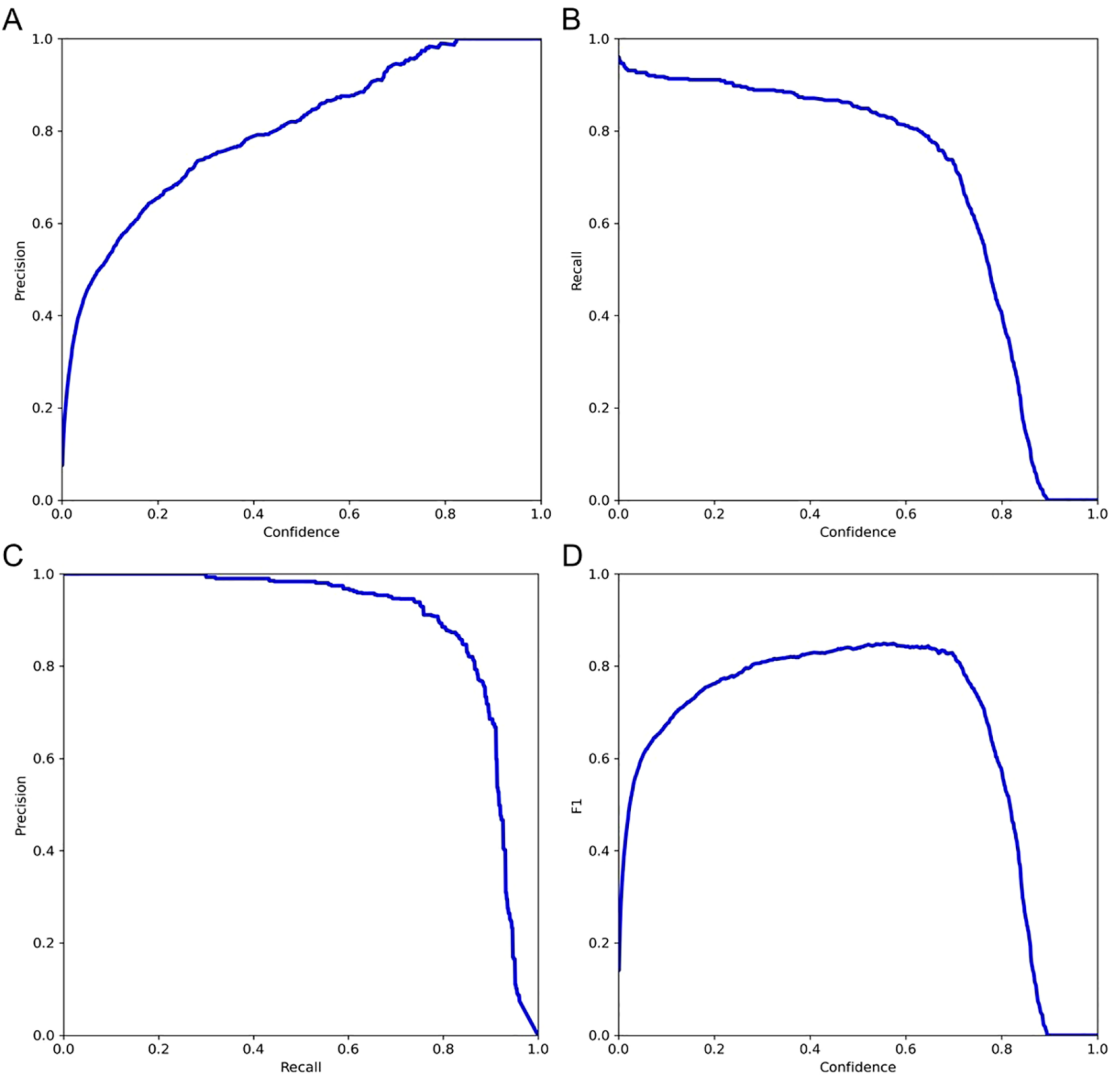
Evaluation of model performance on the testing set. **(A)** Precision versus confidence threshold; **(B)** Recall versus confidence threshold; **(C)** Precision–recall curve; **(D)** F1-score versus confidence threshold.

**Figure 8:**
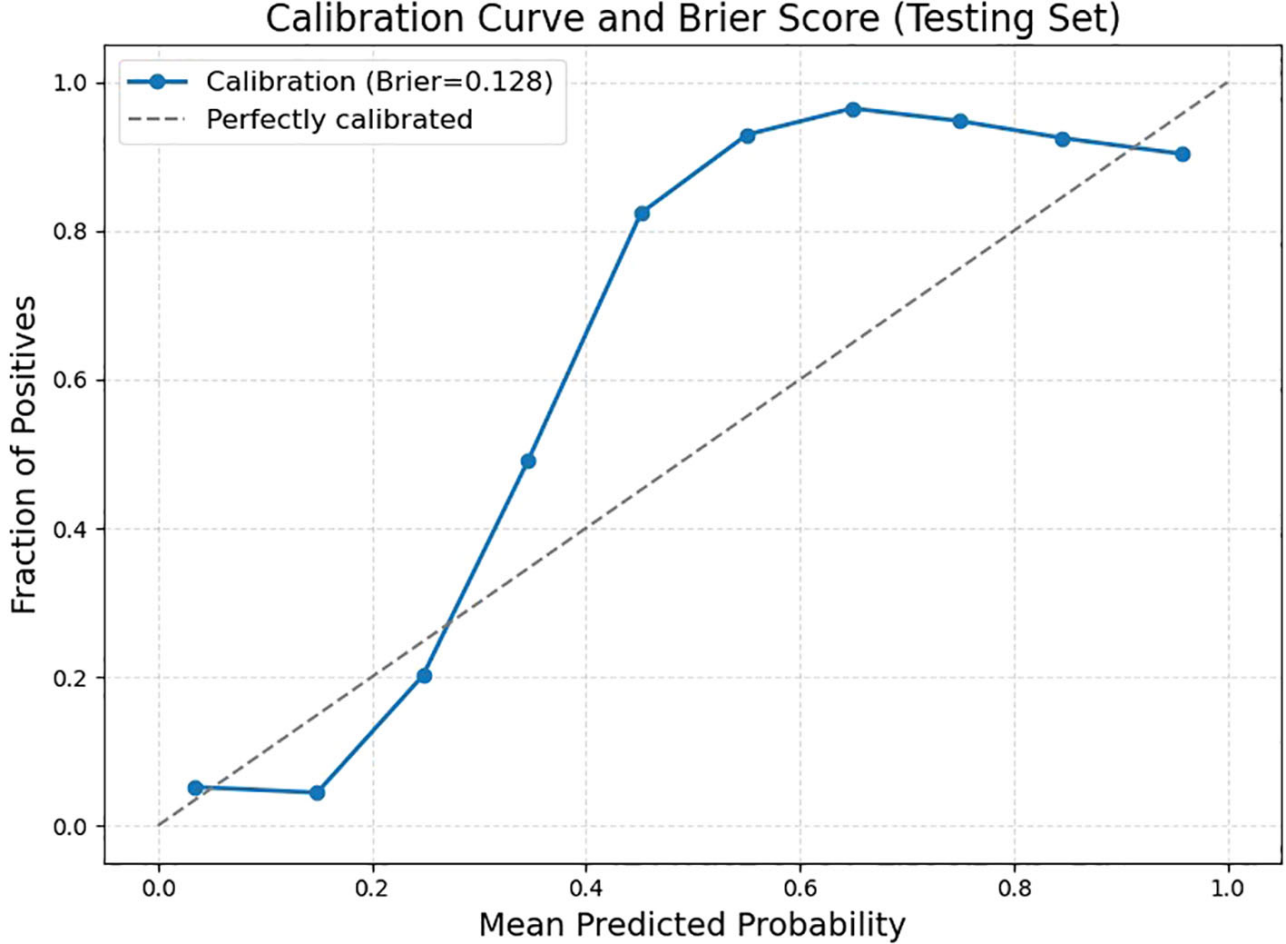
Reliability diagram and Brier score for the testing set.

Further validation on the testing set was performed by examining key performance metrics as functions of the detection confidence threshold (**Figure 7**). The precision-confidence curve (**Figure 7A**) and the recall-confidence curve (**Figure 7B**) show how precision and recall vary inversely with the confidence threshold. Based on the precision–recall curve (**Figure 7C**), the computed AP0.5 is 0.82. The F1‐score—defined as the harmonic mean of precision and recall—was also computed across confidence thresholds (**Figure 7D**) and reaches a maximum value of 0.84 at a threshold of 0.55, which may serve as an optimal operating point. Finally, we evaluated probability calibration using a reliability diagram (**Figure 8**) and quantified it with the Brier score, which was 0.128—indicating that our predicted confidence levels closely match the observed positive‐rate across all thresholds.

To further demonstrate the advancement of our approach, we compared several detection models trained on our dataset and evaluated them on the testing set. All models were trained using our training and validation sets and tested on the same testing set (**Table 4**). Due to the requirement for real-time detection in clinical practice, we used the “S” (small) models, which offer a favorable balance of speed and accuracy. In our experiments, YOLOv7-S (17), YOLOv8-S, and YOLOv9-S (18) achieved AP@0.5 values of 70.4%, 74.7%, and 74.2%, respectively. We selected YOLOv8-S as our model framework due to its high accuracy, and based on this architecture, we introduced further modifications, resulting in an AP@0.5 of 82.0%.

**Table 4:** Comparison of different detection models on the testing set.

Models	AP@0.5 (%)	Parameters (M)	FLOPs (G)	FPS
YOLOv7-S	70.4	11.0	28.1	201
YOLOv8-S	74.7	11.2	28.6	233
YOLOv9-S	74.2	7.1	26.4	242
YOLOv8-S+ DeformConv (ours)	76.2	11.3	29.4	225
YOLOv8-S+ Prototype Loss (ours)	78.6	11.2	28.6	233
YOLOv8-S+ DeformConv+ Prototype Loss (ours)	**82.0**	11.3	29.4	225

This table presents the performance comparison of different detection models evaluated on our testing set. All models were trained and validated using our designated training and validation sets, and final results were obtained on the independent testing set. Model inference was performed on an NVIDIA RTX 2060 GPU with an input image size of 640×640 pixels.Values in bold are emphasized to highlight key results.

### Comparison of diagnostic performance between endometrial thickness and the DL model

To assess the added diagnostic value of DL model, we compared its performance to the classic clinical parameter of endometrial thickness (**Table 5**). The DL model demonstrated significantly superior diagnostic accuracy across multiple metrics. Specifically, the AUC for the DL model was 0.858 (95% CI: 0.800–0.905), significantly higher than that of endometrial thickness (AUC: 0.734 [95% CI: 0.670–0.789], *P* = 0.004).

**Table 5:** Comparison of diagnostic performance between endometrial thickness and the DL model.

Characteristics	Endometrial thickness (95% CI)	Testing set of DL model (95% CI)	*P*-value
AUC	0.734 (0.670 - 0.789)	0.858 (0.800 - 0.905)	0.004
Accuracy	0.750 (0.642 - 0.858)	0.858 (0.760 - 0.956)	0.031
Sensitivity	0.667 (0.531 - 0.803)	0.773 (0.675 - 0.871)	0.043
Specificity	0.786 (0.655 - 0.917)	0.901 (0.803 - 0.999)	0.027
PPV	0.571 (0.421 - 0.721)	0.836 (0.738 - 0.934)	0.009
NPV	0.846 (0.726 – 0.966)	0.867 (0.769 - 0.965)	0.472

DL, deep learning; AUC, area under the curve; CI, confidence interval; PPV, positive predicted value; NPV, negative predicted value.

In addition, the DL model showed higher accuracy (85.8% vs. 75.0%, *P* = 0.031), sensitivity (77.3% vs. 66.7%, *P* = 0.043), specificity (90.1% vs. 78.6%, *P* = 0.027), and positive predictive value (83.6% vs. 57.1%, *P* = 0.009) compared to endometrial thickness. While the negative predictive value was slightly higher for the DL model (86.7% vs. 84.6%), the difference was not statistically significant (*P* = 0.472).

## Discussion

In this study, we developed and optimized a DL model for the identification of EC using ultrasound images, evaluating its performance across three distinct datasets. To the best of our knowledge, this study is among the first to apply a deep learning algorithm to endometrial cancer differentiation using a substantial collection of ultrasound data, building upon and extending findings from related research in adjacent domains. Our model exhibited exceptional performance in distinguishing EC, demonstrating its potential to augment the diagnostic capabilities of less experienced ultrasound physicians. This advancement could significantly enhance both the efficacy and safety of EC detection in primary care settings.

Ultrasound imaging has emerged as the primary tool for initial screening and diagnosis of EC, placing significant demands on the skills of ultrasound physicians in terms of operation, identification, and diagnosis (19). This non-invasive, cost-effective method has become indispensable in gynecological practice (20). However, the current situation in many developing countries, particularly in economically disadvantaged areas, presents a considerable challenge (21). In most primary healthcare institutions in these regions, ultrasound physicians often lack the necessary fundamental skills and knowledge to effectively utilize this technology for EC detection (22). The learning curve for these physicians is typically prolonged, hindered by limited access to advanced training, experienced mentors, and diverse clinical cases (23). This deficiency is particularly concerning given the critical role of early detection in improving EC outcomes.

AI has emerged as a promising solution to bridge the gap in ultrasound expertise, particularly in resource-limited settings (24). The rapid advancements in AI, especially in the field of medical imaging and pattern recognition, offer new possibilities for enhancing diagnostic capabilities in primary care institutions (25). Recent developments in AI applications in modern medicine have been remarkable, particularly in medical imaging and pattern recognition (26). However, to further enhance diagnostic precision, it is crucial to contextualize AI findings within the biological characteristics of tumors. For instance, the structural analysis of HER2 in solid tumors has unveiled novel epitopes that may influence imaging characteristics and AI detection strategies (27). Additionally, insights into tumor metabolism, such as the moonlighting role of enolase-1 in promoting phospholipid metabolism and proliferation, highlight the diverse functional landscapes AI models must implicitly learn from image data (28). Incorporating such domain knowledge could enrich model interpretability and performance in future AI-driven diagnostic systems.

In the specific context of ultrasound and EC detection, DL algorithms have shown promise (29). These algorithms can be trained on large datasets of ultrasound images annotated by experienced specialists, effectively capturing their expertise. The process typically involves:

1. Data Collection: Gathering a diverse set of ultrasound images, including both normal and pathological cases, annotated by expert sonographers (29).
2. Model Training: Utilizing deep learning architectures, such as convolutional neural networks (CNNs), to learn the intricate patterns and features associated with endometrial cancer (30).
3. Validation and Testing: Rigorously evaluating the model’s performance against unseen data to ensure its generalizability and robustness (31).
4. Continuous Learning: Implementing mechanisms for the model to learn from new cases, allowing it to adapt and improve over time (32).

Furthermore, the consistency of baseline clinical features across training, validation, and testing sets, as well as the standardized image annotation protocol, contributed to the robustness and generalizability of the model. The resulting AI model can serve as a powerful tool for both education and clinical practice in primary care settings:

1. Diagnostic Support: The model can provide real-time suggestions or second opinions, helping less experienced physicians make more accurate diagnoses (33).
2. Training Tool: By demonstrating expert-level analysis on a wide range of cases, the AI can accelerate the learning curve for novice ultrasound physicians (34).
3. Continuous Medical Education: The model can be used to create dynamic, case-based learning modules for ongoing physician education (8).

Few previous studies have directly addressed AI-based detection of endometrial cancer via ultrasound. A systematic review reported that among AI applications in gynecologic oncology, only about five studies focused on endometrial cancer, typically employing logistic-regression or radiomics models combining clinical and ultrasound features, with reported areas under the ROC curve (AUC) around 0.90–0.92 in internal validation cohorts (35). For example, Ruan et al. developed a nomogram based on clinical and ultrasound variables (AUC = 0.91, n = 1,837) and Angioli et al. created a similar model (AUC = 0.92, n = 675) (36). More recently, studies using radiomics-based machine learning models have shown validation-set AUC up to 0.90, though typically in narrowly selected patient samples (e.g., postmenopausal bleeding) (36). In contrast, deep DL approaches in ultrasound-based detection of endometrial cancer remain relatively scarce. A multi-center study evaluated DL models for assessing myometrial invasion depth in confirmed EC cases, achieving test-set AUC around 0.81 and demonstrating superior performance compared with radiologists (37–39). While these findings are promising, to our knowledge, no prior study has developed a fully convolutional DL model trained on expert-annotated tumor bounding boxes in ultrasound images encompassing a broader postmenopausal screening population.

Our study extends the existing literature in several meaningful ways. First, we utilized a large, representative cohort of postmenopausal women—including both symptomatic and asymptomatic individuals—enhancing generalizability beyond prior reports focused on high-risk subsets. Second, we implemented a rigorous expert consensus and double-review annotation protocol to generate high-quality tumor region labels across training, validation, and testing sets. Third, our model demonstrated robust performance, with AUC values of 0.89 and 0.87 in internal and external test sets, comparable to or slightly higher than previous radiomics-based approaches, while avoiding reliance on predefined clinical variables (e.g., endometrial thickness thresholds).

By leveraging these AI capabilities, primary care institutions could potentially narrow the expertise gap, providing higher quality ultrasound services even in areas with limited access to specialist knowledge. However, it’s important to note that while AI shows great promise, it should be viewed as a complementary tool to enhance, rather than replace, human expertise. The integration of AI into clinical practice should be done thoughtfully, with ongoing evaluation and adherence to ethical guidelines to ensure patient safety and care quality.

### Limitations

The current investigation has several limitations. Firstly, although multiple internal datasets were used, all data were collected from a single center, which may limit the generalizability and robustness of the model’s performance. The lack of external or multicenter validation restricts our ability to assess the model’s applicability across diverse clinical settings and patient populations. Secondly, although the overall sample size was relatively large, the number of confirmed EC cases may still be insufficient to fully capture the biological and imaging heterogeneity of the disease. Thirdly, variability in ultrasound image quality and scanning techniques among different sonographers may introduce inconsistencies, potentially affecting model reliability. Furthermore, the specialized nature of gynecological ultrasonography requires substantial operator expertise, which may limit widespread implementation of the DL model in routine practice without appropriate training.

In addition, the current dataset did not contain enough stratified patient subgroups (e.g., by histologic subtype, age category, or comorbidity profile) to support meaningful and statistically robust subgroup analyses. As such, we were unable to explore potential performance differences across clinically relevant subpopulations. We recognize this as an important limitation and plan to address it in future studies by expanding the dataset to include a more diverse and balanced patient cohort.

### Future work

To address the limitations identified in this study, our future work will proceed in several directions. First, we plan to conduct external and multicenter validation studies to evaluate the generalizability and robustness of the proposed model across diverse clinical settings and patient populations. Second, we aim to expand our dataset to include a larger number of confirmed EC cases and a broader range of patient subgroups, enabling more comprehensive and statistically meaningful subgroup analyses. Third, to mitigate potential issues related to class imbalance, especially the underrepresentation of certain EC subtypes—we will explore Adaptive Synthetic Sampling (ADASYN) to augment training samples of rare EC subtypes, thereby improving the model’s ability to detect these clinically important but less common categories. Additionally, we will investigate strategies to standardize ultrasound image acquisition and scanning protocols, as well as provide targeted training to sonographers, in order to improve the consistency and reliability of data used for model development.

### Practical implications of the proposed approach

The proposed DL ultrasound model holds substantial practical implications for improving the early detection and diagnosis of EC in primary care settings. First, by enabling real-time, automated interpretation of gynecological ultrasound images, the system reduces dependence on experienced sonographers and mitigates diagnostic variability. This is particularly valuable in resource-limited or rural areas where access to expert clinicians is scarce. Second, the integration of DL into routine ultrasound workflows enhances diagnostic efficiency, allowing more patients to receive timely evaluations and referrals. Third, as the model is trained on a large, well-annotated dataset and demonstrates strong performance metrics across internal validation and test sets, it offers a reliable adjunct to clinical decision-making and has the potential to be deployed as a screening aid or educational tool in primary care environments. Finally, by facilitating early EC detection, this approach may improve long-term outcomes and reduce the burden on tertiary care centers, aligning with broader healthcare goals of early intervention and cost-effective cancer detecting.

## Conclusions

The DL model demonstrated high accuracy and robustness, significantly enhancing the ability to diagnostic assistance for EC through ultrasound in postmenopausal women. This provides substantial clinical value, especially by enabling less experienced physicians in primary care settings to effectively detect EC lesions, ensuring that patients receive timely diagnosis and treatment.

## Data Availability

The raw data supporting the conclusions of this article will be made available by the authors, without undue reservation.
